# Emergent Drug and Nutrition Interactions in COVID-19: A Comprehensive Narrative Review

**DOI:** 10.3390/nu13051550

**Published:** 2021-05-04

**Authors:** Duygu Ağagündüz, Menşure Nur Çelik, Merve Esra Çıtar Dazıroğlu, Raffaele Capasso

**Affiliations:** 1 Department of Nutrition and Dietetics, Faculty of Health Sciences, Gazi University, Emek, Ankara 06490, Turkey; dyt.mensurenurcelik@gmail.com (M.N.Ç.); esracitar@gmail.com (M.E.Ç.D.); 2 Department of Agricultural Sciences, University of Naples Federico II, 80055 Naples, Italy

**Keywords:** COVID-19, drug, nutrition interaction

## Abstract

Coronaviruses are a large family of viruses that are known to cause respiratory tract infections ranging from colds to more severe diseases, such as Middle East Respiratory Syndrome (MERS) and the Severe Acute Respiratory Syndrome (SARS). New Coronavirus Disease 2019 (COVID-19), which led to deaths as well as social and economic disruptions, is an ongoing worldwide pandemic caused by Severe Acute Respiratory Syndrome Coronavirus 2 (SARS-CoV-2). Currently, there is no approved treatment for COVID-19. Hence, only supportive care has been approved by the World Health Organization (WHO) for now. Pharmacological agents used for the adjunctive treatment of COVID-19 following the current literature and clinical experiences include antiviral, anti-inflammatory, and anti-malaria drugs, and other traditional or untraditional treatments. However, it has been reported that the use of these drugs may have some negative effects and comorbidities. Moreover, the current data have indicated that the risk of drug-drug interactions may also be high in polypharmacy cases, especially in elderly people, some comorbidity situations, and intensive care unit (ICU) patients. It is highly possible that these situations can not only increase the risk of drug-drug interactions but also increase the risk of food/nutrition-drug interactions and affect the nutritional status. However, this issue has not yet been entirely discussed in the literature. In this review, current information on the possible mechanisms as well as pharmacokinetic and pharmacodynamic effects of some pharmacological agents used in the treatment of COVID-19 and/or their secondary interactions with nutrition were evaluated and some future directions were given.

## 1. Introduction

Coronaviruses are a large family of viruses that can cause disease in animals or humans. In humans, several coronaviruses are known to cause respiratory tract infections ranging from colds to more severe diseases such as Middle East Respiratory Syndrome (MERS) and the Severe Acute Respiratory Syndrome (SARS). New Coronavirus Disease 2019 (COVID-19) is an ongoing worldwide emergency caused by Severe Acute Respiratory Syndrome Coronavirus 2 (SARS-CoV-2) [1]. The SARS-CoV-2 virus was first reported in a group of patients who developed respiratory symptoms (fever, cough, shortness of breath) in Wuhan Province, China, in late December 2019. As a result of research, it was identified on 13 January 2020. COVID-19, which was declared a pandemic by the World Health Organization (WHO) on 11 March 2020, has and continues to have significant effects in all areas worldwide [2,3]. In the beginning of 2021, several new variants of SARS-CoV-2, such as the South Africa (501Y.V2 or B.1.351), United Kingdom (N501Y or B.1.1.7), and Brazil (P.1) variants, were also detected and have been spreading rapidly worldwide [4].

COVID-19 has often been reported to have a four-stage course. In the first stage, the symptoms are characterized by infection of the upper respiratory tract, while dyspnea and pneumonia begin in the second stage. A cytokine storm is seen in the third stage with a markedly worsened clinical picture, and a hyperinflammatory state associated with this condition is seen in the final stage, with the clinical picture ending in recovery or death [5]. At present, no drug has been validated or approved for treating COVID-19. Therefore, due to the urgent need to identify treatments that could change the course of this pandemic and improve the clinical course for patients with symptoms that may range from mild to critical, research on the use of some old treatment methods that have been repurposed and are being applied adjunctively continues intensively [5,6,7]. Pharmacological agents used in treating COVID-19, as detailed by the current literature and recommendations, include anti-viral, anti-inflammatory, and anti-malarial drugs and other traditional and untraditional treatments and drugs [8]. In this context, although the findings based on strong evidence for treating moderate-severe COVID-19 cases are limited, drugs including remdesivir, teicoplanin, hydroxychloroquine, and ivermectin are among the antiviral drugs that have been used in some countries to control the symptoms of the disease. Tocilizumab can often be considered as a supplementary drug while treating COVID-19 patients presenting with signs of a cytokine storm [6]. The administration of these drugs may have negative effects and comorbidities, however [9]. The US Centers for Disease Control and Prevention reported hydroxychloroquine and chloroquine, specifically approved for the treatment of autoimmune diseases together with the prevention and treatment of malaria, to have potential benefits in preventing and treating COVID-19, but the positive data available at this time do not outweigh the risks of these drugs [10]. In addition, the available data have indicated that the risk of drug-drug interactions may also be high in polypharmacy cases, especially in elderly individuals, in the cases of some comorbidities, and among intensive care unit (ICU) patients [11]. In the presence of these factors, organ dysfunction due to COVID-19 can also change the pharmacokinetics and pharmacodynamics of drugs, which can affect the severity of drug-drug interactions [11].

It is highly possible that these changes may not only exacerbate the likelihood of drug-drug interactions. They may also heighten the risks of food-drug interactions and affect the nutritional status of patients. However, this issue has not yet been comprehensively focused on in the literature. In this review, the possible mechanisms and pharmacokinetic and pharmacodynamic effects of some pharmacological agents used in treating COVID-19 or alleviating its symptoms are preliminarily examined in light of their secondary interactions with nutrition.

## 2. COVID-19 Treatment

No treatment for COVID-19 has received approval at the time of writing. For this reason, the WHO currently only approves supportive care. At the same time, throughout the course of the pandemic, clinicians and researchers have continued to experiment with a variety of virus-based and host-based therapeutics [12]. Although estimates of the number of clinical trials that are currently underway vary, it is generally thought to be about 800 clinical trials [13].

As the safest method for now, individual risk management is very important for minimizing infection risk and reducing disease severity levels for patients who have been diagnosed with SARS-CoV-2 infection. As a result of the bidirectional interactions existing among nutrition, infection, and the immune system, a change in one of these components affects the others [14]. In addition to age-related micronutrient deficiency, an individual’s pre-existing nutritional status will have notable effects on his or her risk of infection as well as the clinical course and outcomes. Therefore, maintaining adequate macronutrient and micronutrient balances is a valuable strategy for preventing COVID-19 infection [15]. However, it should be kept in mind that nutrition alone will not be sufficient in the presence of various metabolic disorders, demographic variables, or lifestyle patterns including advanced age, comorbidities, regular exposure to environmental toxins, or tobacco usage [14].

In supportive treatment of COVID-19, factors such as the age of the patient, the severity of the clinical characteristics, the presence of indicators of poor prognosis, and the presence of other diseases are all important [16]. Although some vaccines have been investigated for their safety and efficacy in treating or preventing the disease, Phase 3 studies are still ongoing or have been newly approved. These vaccines are not yet sufficiently widespread. For this reason, drugs with antiviral activity are currently widely used for supportive care [2,17]. Frequently used supportive treatments in line with the recommendations of guidelines all over the world have four main objectives: (i) stop the virus from being able to enter cells, (ii) reduce or inhibit the replication of the virus, (iii) suppress the increased and uncontrolled inflammation response caused by the disease, and (iv) ensure the neutralization of the virus with immune plasma treatments containing antibodies against the virus obtained from patients who have recovered from the disease [18].

The toxicity and the efficacy of these drugs should be considered simultaneously in treating these patients, and treatment agents must be selected considering each individual patient’s clinical picture [19]. Different algorithms are observed in different countries for treating COVID-19 infections. For example, the supportive treatments included in the algorithms recommended by the Ministry of Health of the Republic of Turkey are applied for each patient diagnosed regardless of the symptoms. The drugs included in these algorithms are mainly hydroxychloroquine, favipiravir, and azithromycin. In some cases, drugs such as lopinavir/ritonavir, tocilizumab, and anakinra may also be used if macrophage activation syndrome indications are observed. Another agent used for treating COVID-19 in other countries is remdesivir. At the same time, empirical treatments are being applied in the clinical picture of cytokine storms in the later stages of the disease. Interleukin (IL)-6 inhibitors and steroids are expected to be effective [2].

**Figure 1:**
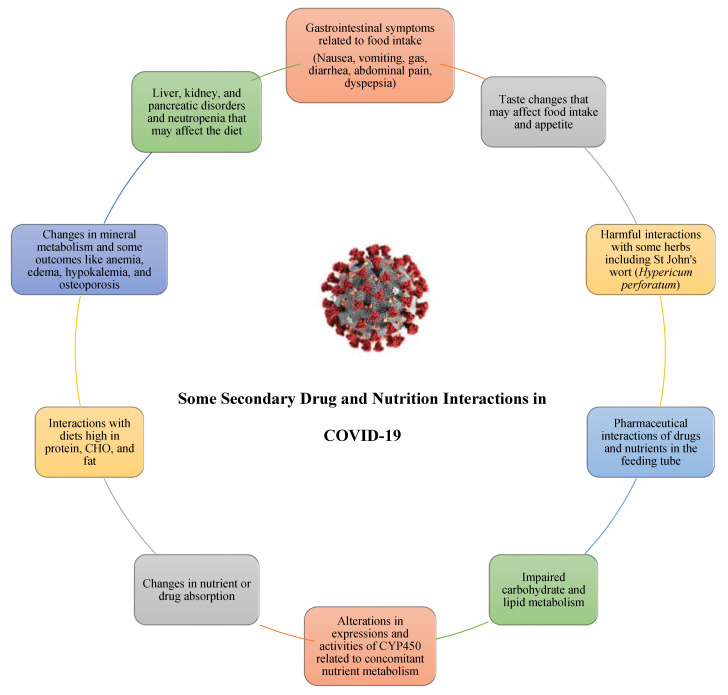
An overview of potential secondary nutritional interactions of drugs currently being used in treating COVID-19.

In the general course of COVID-19, the eating behaviors of the patients change and their nutritional status may be affected. It was reported that even individuals who are not infected have an unhealthy diet and lifestyle during self-quarantine or isolation [20]. In infected patients, considering the gastrointestinal effects of the disease, the appetite of some patients decreases, and especially elderly patients and ICU patients may be unable to fulfill their nutritional aims with only oral diets, possibly even requiring enteral-parenteral nutritional support [21]. In addition, pharmacological drugs used as supportive care can result in food-drug interactions and affect the nutritional status of patients both acutely and chronically. In Figure 1, the interactions with nutrition of some potential drugs utilized in treating COVID-19 are summarized.

### 2.1. Anti-Viral Drugs

The majority of drugs being used in treating SARS-CoV-2 infection were selected in light of the experience obtained from treatment protocols for SARS or MERS [19]. There is no specific antiviral agent proven in terms of safety and efficacy that has completed randomized controlled clinical trials for COVID-19. Antiviral drugs administered in the immediate timeframe following symptom onset may facilitate a reduced viral load and, accordingly, reduce the infectiousness of the virus in patients’ respiratory secretions. Considering that the SARS-CoV-2 viral load peaks in sputum within 5 or 6 days after symptom onset while viral shedding continues for 14 days, these drugs have advantages, such as shortening the treatment duration, improving prognosis, and reducing viral shedding and spread [22]. The most common clinical abnormalities seen with antiviral therapy have been reported as diarrhea, constipation, and decreased food intake [23]. Table 1 summarizes some antiviral drugs and additional treatments used in treating COVID-19 together with their mechanisms of action, some common adverse effects, and recommendations for administrations.

#### 2.1.1. Hydroxychloroquine/Chloroquine

Traditionally, hydroxychloroquine/chloroquine has been particularly indicated for prevention and treatment of malaria. Additionally, it is useful in the treatment of rheumatoid arthritis, erythematosus and systemic lupus erythematosus, and chronic discoid lupus [29].

#### 2.1.2. Mechanism of Action

Hydroxychloroquine/chloroquine seems capable of blocking the virus’s entrance into cells by inhibiting host receptors’ glycosylation, endosomal acidification, and proteolytic processing. Immunomodulatory effects are also exerted as a result of impaired cytokine production and inhibited autophagy and lysosomal activities within host cells [30,31].

Since hydroxychloroquine has previously been widely used with different indications and its safety has been demonstrated in humans, it was commonly used around the globe, including in Turkey, for treating potentially fatal cases of COVID-19, and it is still being used in many countries. However, in the eight months following the onset of the outbreak, published studies on the effect of hydroxychloroquine/chloroquine in cases of SARS-CoV-2, its place in COVID-19 treatment processes, and, in particular, its undesirable cardiotoxic effects in cases of COVID-19 have necessitated a reevaluation of hydroxychloroquine’s role in treating this disease [32]. The initial positive outlook on hydroxychloroquine/chloroquine has been replaced by the “recommendation not to use in treatment” due to the inability to demonstrate an effect of the drug on mortality during the pandemic and its potential toxicity [33].

#### 2.1.3. Pharmacokinetics and Pharmacodynamics

Hydroxychloroquine/chloroquine disperses throughout the body upon oral administration. While hydroxychloroquine absorption varies (about 70%), chloroquine displays absorption that is fast and almost entirely complete. These drugs are both moderately (about 40%) bound to plasma proteins [34,35]. Cytochrome P450 (CYP) enzymes hold the responsibility for catalysis of the dealkylation of hydroxychloroquine into pharmacologically active metabolites, while hydroxychloroquine/chloroquine is metabolized in the liver via the CYP3A, CYP2D6, and CYP2C8 systems [35]. While chloroquine’s elimination half-life is 4 to 5 days, that of hydroxychloroquine is approximately 40 days. Moreover, both are excreted renally and their excretion increases with urinary acidification.

Approximately 40–60% of hydroxychloroquine/chloroquine is metabolized from the kidneys, 8–25% is excreted through feces, about 5% is excreted through the skin, while 25–45% remains stored in lean tissues for a long time. A few years after administration, a small amount of the drug still remains in plasma, urine, and erythrocytes. As a result of kidney or liver dysfunction, an increase in the retained drug level and a risk of adverse effects may occur with the decrease of hydroxychloroquine excretion [36].

#### 2.1.4. Adverse Effects and Nutrition Interactions

Hydroxychloroquine is generally well tolerated. Drug absorption is not impacted by food intake [36]. In the table, regarding the administration of COVID-19 drugs in tablet form prepared by the Liverpool Drug Interaction Group, for patients with swallowing difficulties (dysphagia), it is stated that the crushing of chloroquine-containing tablets is not recommended, but, if crushed, they can be given to patients by mixing with honey, jam, pasteurized yogurt, or similar foods [37]. The adverse effects reported most commonly are disorders of the gastrointestinal system including nausea and diarrhea, anorexia, abdominal pain, vomiting, and dermatological reactions such as hair loss, itching, pigmentation, and skin rashes. These adverse effects usually disappear with a dose reduction and rarely require discontinuation of treatment [38]. However, hydroxychloroquine is known as a causative agent for severe hypoglycemia, with possible life-threatening loss of consciousness in patients treated/not treated with anti-diabetic drugs. It is important to monitor blood glucose levels in patients presenting with clinical symptoms, suggesting hypoglycemia in the course of hydroxychloroquine treatment, and general treatment should be reviewed if necessary. Caution is similarly warranted for patients with quinine intolerance, glucose-6-phosphate dehydrogenase deficiency, or porphyria cutanea tarda, which may be inflamed with hydroxychloroquine, as well as psoriasis, as this drug apparently increases the risks of skin reactions. Individuals with rare hereditary galactose intolerance, glucose-galactose malabsorption disease, and Lapp lactose deficiency are not eligible for use of this drug [37].

#### 2.1.5. Favipiravir

Favipiravir is a pro-drug of ribofuranosyl-5′-triphosphate, a purine nucleotide previously known as T-705. Most preclinical data on favipiravir were obtained from studies on influenza and Ebola, but it is known to exhibit significant activities against other RNA viruses as well [39].

#### 2.1.6. Mechanism of Action

Favipiravir is an RNA-dependent RNA polymerase inhibitor. As a pro-drug, it is a purine base analog undergoing conversion to active favipiravir ribofuranosyl-5B-triphosphate (favipiravir-RTP) as a result of intracellular phosphoribosylation. It is an inhibitor of the RNA-dependent RNA polymerase (RdRp) found in RNA viruses, acting selectively and with notable potency [40]. Favipiravir-RTP acts with the selective inhibition of RNA polymerase, preventing the replication of the viral genome. Various hypotheses have been proposed regarding the interaction of favipiravir-RTP with RdRp. Previous studies have concluded that favipiravir-RDP prevents RNA spiral elongation and viral proliferation when incorporated into a newly formed RNA spiral [16]. Favipiravir is distinguished from other antivirals by its direct inhibition of viral replication and transcription and by its specific mechanism of action targeting viral RNA polymerase [41].

#### 2.1.7. Pharmacokinetics and Pharmacodynamics

Favipiravir has a high bioavailability of approximately 94% and a protein binding capability of 54%. After a single dose, it reaches its maximum concentration in 2 h. Its half-life is quite short, ranging between 2.5 and 5 h, which leads to accordingly fast renal elimination in a hydroxylated form. After administering multiple doses, both the peak time and the half-life are increased. Elimination can be mediated by aldehyde oxidase and partially by xanthine oxidase. The pharmacokinetics of favipiravir are dependent on both time and dose. The metabolism of the parent drug occurs in the liver, driven primarily by aldehyde oxidase and partly by xanthine oxidase, yielding an inactive oxidative metabolite, T-705M1, which is excreted by the kidneys [42]. While it is not metabolized by the CYP system, it does inhibit CYP2C8, which is a component of the cytochrome enzyme system. Therefore, caution should be used when administering drugs that are metabolized by the CYP2C8 system [42,43].

#### 2.1.8. Adverse Effects and Nutrition Interactions

In general, favipiravir is tolerated well, but its common adverse effects may be listed as gastrointestinal adverse effects, such as mild to moderate diarrhea, nausea, increased gas, elevated uric acid, decreased neutrophil counts, and increased aspartate aminotransferase (AST), alanine transaminase (ALT), and blood triglycerides [40,44].

Favipiravir undergoes metabolization to its inactive metabolite M1 with aldehyde oxidase and xanthine oxidase to finally be excreted in the urine. Favipiravir and M1 both inhibit organic anion transporters 1 and 3 (OAT1 and OAT3), which facilitate kidney excretion of uric acid. Furthermore, M1 increases uric acid re-uptake in the proximal renal tubules via urate transporter 1 (URAT1). It is thought that this is why favipiravir is able to reduce uric acid excretion via urine and cause elevated blood uric acid levels. These heightened levels of uric acid return to values within the reference ranges upon discontinuing the drug. However, it should be kept in mind that this action of favipiravir may have clinical significance in patients with histories of gout, renal dysfunction (increased blood concentrations of M1), or hyperuricemia as well as in patients simultaneously using other drugs that trigger elevated levels of blood uric acid [45]. Favipiravir tablets are recommended to be taken orally while fasting. Studies have reported no significant difference in favipiravir administration while fasting, with food, or 30 min after eating. It is recommended to administer it according to the prospectus [44].

#### 2.1.9. Remdesivir

Remdesivir was recently described as an antiviral drug possessing great promise against a considerable variety of RNA viruses including SARS and Middle East respiratory syndrome coronavirus 5 (MERS-CoV-5) in models established in mice, cell cultures, and non-human primates [46]. Remdesivir is a pro-drug of the adenosine nucleotide analog, which is able to inhibit viral RNA polymerase and is metabolized to the intracellular adenosine triphosphate analog. It is a new antiviral drug possessing antiviral activities against various RNA viruses [47,48].

#### 2.1.10. Mechanism of Action

Remdesivir is a nucleoside analog used to inhibit the action of RNA polymerase. It prevents the addition of nucleotides to RNA, resulting in RNA transcription termination [49]. With the early termination of RNA transcription, viral replication decreases and pulmonary function improves with the reduction of the lungs’ viral load [50,51].

#### 2.1.11. Pharmacokinetics and Pharmacodynamics

While remdesivir has 80–90% protein binding, its metabolite, GS-441524, has much lower protein binding levels (<20%) in plasma. Results obtained from healthy human donors definitively revealed the metabolizing of remdesivir by CYP enzymes (CYP2C8, CYP2D6, and CYP3A4). However, specific data on the metabolism of GS-441524 are not yet available. Remdesivir and GS-441524 have half-lives of approximately 0.89 and 25 h, respectively. The majority of remdesivir is excreted through the urine (about 74%) [52].

#### 2.1.12. Adverse Effects and Nutrition Interactions

Adverse effects such as gastrointestinal symptoms (nausea and/or vomiting) and aminotransferase elevations have been reported among some patients using remdesivir, and daily liver and kidney function tests are recommended for performance [53,54].

#### 2.1.13. Lopinavir-Ritonavir

Lopinavir/ritonavir (Lop/r), combined as a single oral agent that received approval from the US Food and Drug Administration (FDA) for HIV treatment, has demonstrated in vitro activity against other coronaviruses by inhibiting three chymotrypsin-like proteases [55,56]. Lopinavir was combined with ritonavir as a pharmacokinetic enhancer [57]. Ritonavir’s cytochrome P450 inhibitory effect was found to prolong both lopinavir’s half-life and its protease inhibitory effect on HIV replication. Additionally, in vitro research has revealed that Lop/r in combination may inhibit coronavirus replication [15].

To date, reports on the usage of Lop/r for COVID-19 have been merely case reports or retrospective, non-randomized cohort studies that are small in size. Therefore, the direct effects of Lop/r on such treatment still await full clarification [58,59].

#### 2.1.14. Mechanism of Action

Lopinavir is effective in the inhibition of replication of the virus because it blocks the main protease of SARS-CoV-2. Researchers have reported that Lop/r inhibits SARS-CoV-1 in vitro, and the two drugs work synergistically with each other [57,60].

Lop/r has shown activity against the coronavirus in in vitro settings. The majority of similar studies concluded that lopinavir inhibits SARS-CoV, with the EC50 value of the drug being found to fall within acceptable ranges. In particular, lopinavir demonstrated antiviral effects in Vero E6 cells upon exposure to the SARS-CoV-2 virus [47].

#### 2.1.15. Pharmacokinetics and Pharmacodynamics

Lopinavir is bound to plasma proteins at rates of approximately 98–99%. It undergoes hepatic metabolism thanks to CYP3A4 as a result of drug interactions with all CYP3A4 inhibitors and inducers. The elimination half-life of this drug is from 5 h to 6 h with a peak time of approximately 4 h. The excretion of the drug is mainly through the feces (approximately 83%) and the remainder is excreted through the urine. Due to the low rate of elimination by the kidneys, dose adjustments are not necessary for patients with renal diseases and they can use this drug safely [34].

Ritonavir is similarly bound to plasma proteins at rates of approximately 98–99%, undergoing metabolism in the liver via CYP2D6 and CYP3A4. Ritonavir, when administered in low doses together with lopinavir, acts as a pharmacokinetic enhancer [61]. Its elimination half-life ranges between 3 and 5 h. The absorption of this drug and its oral bioavailability both vary when patients are unfed or fasting. These values, together with peak levels, increase notably with food intake. However, foods will delay the peak time, which occurs at 2 h in fasting states, or 4 h in cases of fullness. Since the percentage of ritonavir elimination from the kidneys is very low, it does not necessitate dose adjustments for patients with concomitant diseases of the kidneys [34].

#### 2.1.16. Adverse Effects and Nutrition Interactions

This drug may cause diarrhea, nausea, vomiting, liver disorders, pancreatitis, hypercholesterolemia, hypertriglyceridemia, fatigue, skin rashes, arrhythmia, hypersensitivity, neutropenia, and thrombocytopenia [54,62,63].

Upon administration of lopinavir in liquid or capsule form in the lopinavir/ritonavir formulation, its bioavailability may increase significantly with the concomitant consumption of foods with moderate or high fat contents. As a result, Lop/r should be administered with meals that are moderately high or high in fat [64].

Patients using Lop/r must be told to avoid using any products supplemented with St. John’s wort (Hypericum perforatum) among their ingredients, as it might trigger a decrease in the plasma concentration of the drug or a decrease in clinical effects [65].

#### 2.1.17. Umifenovir

Umifenovir, also known by the brand name Arbidol, is an indole-derived antiviral treatment approved for the prevention and treatment of influenza in both China and the Russian Federation, and interest in its use in COVID-19 treatment is increasing daily [66,67].

#### 2.1.18. Mechanism of Action

Umifenovir is a new and promising antiviral agent that targets S protein/ACE-2 interaction and it has a mechanism of action that inhibits the viral envelope’s membrane fusion. Umifenovir acts by making any contact of the virus with target host cells impossible [25,68].

#### 2.1.19. Pharmacokinetics and Pharmacodynamics

As an indole derivative, umifenovir is poorly soluble in water, which affects its bioavailability and pharmacokinetics. Upon administration by an oral route, umifenovir is rapidly distributed into the body’s tissues and organs, reaching maximum concentrations in plasma within 1–1.5 h. It is a drug that is metabolized in the liver. Umifenovir can undergo various metabolic processes, including oxidation in the S-region, N-demethylation, glucuronidation, and conjugation at the 5-hydroxy part. However, the possible antiviral actions of umifenovir metabolites remain undiscovered [69].

Approximately 40% of the total administered umifenovir dose is eliminated unchanged within a time span of 48 h. The primary excretion path is feces (38.9%) and much less is excreted in urine (0.12%) [70].

#### 2.1.20. Adverse Effects and Nutrition Interactions

Umifenovir is generally tolerated well by its users. Mild adverse effects in the gastrointestinal system, including nausea, diarrhea, and stomach pain, and mild to moderate ALT elevations have been reported in some patients [25,67].

#### 2.1.21. Oseltamivir

Oseltamivir was approved for the treatment of influenza A and B. On the other hand, it is not effective in the treatment of COVID-19 and is not currently recommended for that purpose [25]. Accordingly, the Ministry of Health of the Republic of Turkey does not recommend oseltamivir for COVID-19. For patients with clinical findings consistent with influenza, however, it may be useful [71].

#### 2.1.22. Mechanism of Action

In human patients, oseltamivir targets neuraminidase on the influenza virus’s surface to prevent the influenza virus from spreading within the body [72]. Neuraminidase glycoprotein facilitates viral release from infected cells and its penetration into the airways [73].

#### 2.1.23. Pharmacokinetics and Pharmacodynamics

Oseltamivir is a pro-drug taken as oseltamivir phosphate and it rapidly metabolizes into the active form, oseltamivir carboxylate [74]. Following oral intake of oseltamivir phosphate, it undergoes rapid absorption from the gastrointestinal tract with subsequent conversion by hepatic esterases into oseltamivir carboxylate (80%). This active metabolite can be detected in the plasma within 30 min and its concentration reaches near maximum levels within 3–4 h [74,75]. Oseltamivir shows less binding to plasma proteins. Its metabolism occurs independently of the CYP and glucuronidase systems [71]. Oseltamivir phosphate and oseltamivir carboxylate are mainly excreted by the renal route, even though small amounts of both compounds (20% of the oral dose) also get excreted via the fecal route [74]. Oseltamivir phosphate has an elimination half-life of 1–3 h. Upon oral ingestion, its plasma concentrations decrease rapidly. In contrast, oseltamivir carboxylate has a longer elimination half-life within the range of 6–10 h and its concentration decreases over a longer period of time [74,75].

#### 2.1.24. Adverse Effects and Nutrition Interactions

Oseltamivir is a precursor drug with good bioavailability in oral use. Taking it with foods does not affect plasma density but may delay reaching the highest density [76].

While nausea, vomiting, encephalitis, and encephalopathy have been reported as the most common adverse effects, anemia may occur less rarely than 1%. Hypothermia impaired liver function tests, hepatitis, gastrointestinal bleeding, hemorrhagic colitis, and diabetes exacerbation that are among the adverse effects of unknown frequency [54]. Taking oseltamivir with food significantly reduces gastrointestinal system-related adverse effects [73].

#### 2.1.25. Ribavirin

Ribavirin is a guanine analog antiviral drug with the ability to successfully inhibit viral RdRp. Due to its activity against other coronaviruses, it was thought to be used for COVID-19 treatment [77].

#### 2.1.26. Mechanism of Action

Five different mechanisms are suggested as explanations for ribavirin’s antiviral properties, comprising direct mechanisms (RNA closure, inhibition of RNA polymerase, and fatal mutagenesis) and indirect mechanisms (immunomodulatory effects and inosine monophosphate dehydrogenase inhibition) [78].

#### 2.1.27. Pharmacokinetics and Pharmacodynamics

Ribavirin is an antiviral drug with hepatic metabolism that has an oral bioavailability of approximately 64%. While its elimination half-life is 24 h among healthy individuals, it can increase to 44 h in those with preexisting chronic hepatitis C infection. Therefore, ribavirin must not be administered to individuals suffering from hepatic impairments, particularly those of Child-Pugh class B and C, due to its long half-life and the potential for toxicity in cases of overdoses. After oral administration, the time to reach the highest level is between 2 and 3 h. Ribavirin can be excreted via both feces and urine. Since it is eliminated by the kidneys, adjustments to the prescribed dosage are important in patients with renal diseases [79]. Ribavirin distribution in the erythrocytes, which can cause ribavirin-induced anemia, can extend from about 16 to 40 days [80].

#### 2.1.28. Adverse Effects and Nutrition Interactions

It has been reported that this drug can lead to severe hemolytic anemia, typically occurring within the first 1–2 weeks of its administration. For this reason, hemoglobin and hematocrit control is recommended before treatment and at the second and fourth weeks of treatment. Less rarely, symptoms of weakness and nausea have also been reported [54].

#### 2.1.29. Nitazoxanide

Nitazoxanide is an antiprotozoal with FDA approval currently used in the treatment of both cryptosporidium and giardia. Furthermore, it has proven to exert extensive antiviral activities and it was approved in some countries to treat noroviruses and rotaviruses [81]. Nitazoxanide, known as an anthelmintic agent, has a relatively positive safety profile together with its extensive antiviral activities. Promisingly, its in vitro antiviral activities against both MERS and SARS-CoV-2 were also reported [82,83].

#### 2.1.30. Mechanism of Action

Nitazoxanide’s antiviral activities are associated with host-regulated pathways during viral replication. This antiviral agent triggers hereditary antiviral mechanisms via cytoplasmic RNA and type I interferon pathways [83,84]. Nitazoxanide regulates natural antiviral mechanisms by extensively increasing cytoplasmic RNA detection as well as type I IFN pathways [85].

#### 2.1.31. Pharmacokinetics and Pharmacodynamics

Plasma esterases hydrolyze nitazoxanide in the blood to tizoxanide as the desacetyl derivative, or desacetyl nitazoxanide. This derivative is an active metabolite in vivo and is the sole form that can be measured in the plasma. Upon oral intake of nitazoxanide, the plasma concentration of tizoxanide reaches its maximum within 1 to 4 h. Tizoxanide binds to plasma proteins at extremely high levels (199%). The elimination half-life in the urine is 7.3 h. It can be readily found in plasma, bile, feces, and urine [86]. Nitazoxanide, on the other hand, undergoes absorption in the gastrointestinal system and roughly one-third of the oral dose is shed via urine, while two-thirds is excreted through the feces [87]. While metabolism studies conducted in vitro concluded that tizoxanide had no noteworthy inhibition effects on CYP enzymes, it has been reported that care should be taken in its administration to patients with impaired liver or kidney functions, as the pharmacokinetics of nitazoxanide have not been thoroughly studied [88].

#### 2.1.32. Adverse Effects and Nutrition Interactions

To a large extent, the adverse effects of nitazoxanide are mild, have short durations, and typically involve the gastrointestinal tract. In one study, diarrhea, oropharyngeal pain, abdominal pain, and vomiting were reported as common adverse effects [81,89]. Nitazoxanide undergoes effective absorption from the gastrointestinal tract following its administration by the oral route. When the drug is taken with food, its absorption approximately doubles [86].

### 2.2. Anti-Inflammatory Agents

Nonsteroidal anti-inflammatory drugs (NSAIDs) mainly exert inhibitory effects on cyclooxygenase (COX) by preventing arachidonic acid’s production of prostaglandin [90]. NSAIDs inhibit COX enzymes in a nonselective manner and reduce inflammation by reversing cyclooxygenase-1 (COX-1) and cyclooxygenase-2 (COX-2) [91]. This drug group, which has analgesic, antipyretic, and anti-inflammatory effects, is not only effective in inflammatory diseases like rheumatoid arthritis because of exerting anti-inflammatory effects. It is also indicated for osteoarthritis, soft tissue damage, renal colic, and postoperative pain [92].

#### 2.2.1. Ibuprofen

Ibuprofen from the NSAID group was the pioneering drug among the propionic acid derivatives, having been first marketed in 1969 [93]. Ibuprofen is a recommended antipyretic and analgesic drug, even in the pediatric population [94]. Concerns about the usage of ibuprofen during the COVID-19 pandemic began with the French Ministry of Health stating on 14 March 2020, where the consumption of anti-inflammatory drugs might be an aggravating factor for infection [95]. However, subsequent epidemiological findings have not supported the idea that ibuprofen exacerbates the likelihood of infection in COVID-19 patients [96,97].

#### 2.2.2. Mechanism of Action

Ibuprofen is a nonselective inhibitor of COX-1 and COX-2. Although its anti-inflammatory properties are weaker than those of other members of the NSAID family, its analgesic and antipyretic properties are significant. These are dependent on the inhibitory effects on COXs, which play roles in synthesizing prostaglandins [93].

#### 2.2.3. Pharmacokinetics and Pharmacodynamics

Ibuprofen, which is mostly taken orally, can also be applied topically, intraocularly, intravenously, intramuscularly, and rectally [98]. Ibuprofen undergoes rapid absorption upon its oral administration and it usually achieves its peak serum or plasma levels within 1.5 to 2 h [99]. Ibuprofen is subjected to enantiomeric inversion and hepatic oxidative metabolism by CYP2C9 and undergoes urinary excretion as a glucuronide-conjugate metabolite [100].

#### 2.2.4. Adverse Effects and Nutrition Interactions

Major adverse effects include effects on the gastrointestinal tract, renal system, and clotting. The most commonly reported adverse effects are seen in the gastrointestinal system, including dyspepsia, nausea, and vomiting [93]. It is also associated with inflammatory bowel disease exacerbations and may contribute to the progression of the disease [101]. Rare adverse effects of ibuprofen have been reported, such as dizziness, headaches, skin rashes, blurred vision, thrombocytopenia, and, in some patients, fluid retention, toxic amblyopia, and edema [93].

Considering the interaction of ibuprofen with nutrition, some studies on nutrients have been conducted to date and their effects on drug absorption have been examined. For example, it was stated that daily ibuprofen doses should be reduced in people with high consumption of Cola, as the absorption of ibuprofen increases with consumption of that beverage [102]. Garba et al. found that an extract of the fruit Tamarindus indica raised the bioavailability levels of ibuprofen dramatically [103]. Similarly, concomitant use of caffeine with ibuprofen has been shown to have a stronger effect than the use of ibuprofen alone. However, prolonged use of this combination may lead to a risk of adverse reactions in the central nervous system and excessive analgesic abuse syndrome when the taken dose is high [104].

According to the results of a study showing that ibuprofen-like activity is exerted by extra virgin olive oil, the olive oil specifically generates cyclooxygenase-inhibiting enzyme activity. As a result, daily consumption of 50 g of extra virgin olive oil that contains up to 200 µg of oleocanthal per milliliter will correspond to daily ibuprofen intake of up to 9 mg, on average, considering that 60–90% of it is absorbed. This dose corresponds to roughly 10% of the recommended ibuprofen dosage to relieve pain in the adult population [105].

#### 2.2.5. Indomethacin

Indomethacin, first approved in the United States in 1965, is an NSAID belonging to the methylated indole class and has analgesic, antipyretic, and anti-inflammatory properties similar to other members of the NSAID family. Indomethacin is a powerful analgesic that may be utilized in a wide range of therapeutic applications [106]. Among NSAIDs known to cross the blood-brain barrier, indomethacin exhibits a significant effect in treating headaches by passing through the blood-brain barrier at the highest level as compared to naproxen and ibuprofen [107].

#### 2.2.6. Mechanism of Action

Indomethacin is a potent and nonselective time-dependent COX-1 and COX-2 inhibitor. It exhibits approximately 15 times higher selectivity for COX-1 than COX-2 [108].

#### 2.2.7. Pharmacokinetics and Pharmacodynamics

Indomethacin undergoes rapid absorption from the gastrointestinal tract and its bioavailability is approximately 100%. The peak plasma concentrations are seen to occur between 0.9 ± 0.4 and 1.5 ± 0.8 h in the fasting state following oral administration. It undergoes metabolism in the liver via conjugation with glucuronic acid. O-desmethylation and N-deacylation also happen at significant rates. The disappearance of indomethacin from plasma occurs with a biphasic pattern. There is a half-life of 1 h for the first phase, while the second phase has a half-life ranging from 2.6 to 11.2 h. This relatively wide range is assumed to be the result of differences occurring among individual patients in the drug’s enterohepatic circulation [90].

#### 2.2.8. Adverse Effects and Nutrition Interactions

Indomethacin can have many adverse effects associated with the cardiovascular system, gastrointestinal system, and nervous system, as well as hematological, dermatological, renal, and hepatic effects [90]. Regarding the gastrointestinal system, NSAIDs are aggressive factors that cause damage to the gastrointestinal mucosa [109]. Long-term usage has been linked to severe gastropathy, which might result from gastric mucosal cell apoptosis. Indomethacin also causes excessively high levels of gastric acid secretions and the production of reactive oxygen species (ROS) while preventing regeneration of mucosal cells [110]. In a study conducted along these lines, camellia oil, which is an edible oil type widely used in Asia, possesses powerful antioxidant and anti-inflammatory properties and is a functional dietary oil with the ability to prevent both oxidative damage and inflammation in the gastrointestinal mucosal damage caused by indomethacin [111]. In another study, the ability of black tea to heal indomethacin-induced stomach ulcers with an antioxidant effect was demonstrated [112].

When indomethacin is taken with nutrients, its absorption decreases, being particularly delayed with the consumption of diets high in carbohydrates, protein, and fats, respectively. However, its bioavailability is not impacted in this regard. Similarly, when taken with antacids containing aluminum and magnesium hydroxide, peak concentrations in plasma experience a slight decrease, but this is not thought to have clinical relevance [90].

### 2.3. Interleukin Inhibitors

Interleukins are cytokines playing key roles in immunoregulation and inflammation by the promotion of the regulation, activation, proliferation, and migration of leukocytes. For this reason, interleukin inhibitors can be used to treat immunological diseases, such as ankylosing spondylitis, rheumatoid arthritis, psoriatic arthritis, psoriasis, and inflammatory bowel disease [113]. Interleukin inhibitors (tocilizumab or anakinra) can also be used in patients who have developed macrophage activation syndrome during COVID-19 when an adequate response to glucocorticoid treatments has not been achieved [114].

#### 2.3.1. Tocilizumab

Tocilizumab is a recombinant humanized monoclonal antibody with the ability to inhibit the interleukin (IL)-6 receptor [115]. It is mainly used for treating rheumatoid arthritis, systemic polyarticular juvenile idiopathic arthritis, and juvenile idiopathic arthritis [116].

#### 2.3.2. Mechanism of Action

Tocilizumab forms bonds selectively and competitively with both membrane-bound and soluble IL-6 receptor, thereby, inhibiting IL-6 from binding to its receptor. Inhibition of the whole receptor complex also blocks IL-6 signal transmission to inflammatory mediators calling B and T cells [73].

#### 2.3.3. Pharmacokinetics and Pharmacodynamics

Tocilizumab binds to the IL-6 receptor dose-dependently, saturating the receptor at concentrations of about 0.1 µg/mL. This drug is also able to competitively inhibit the binding of IL-6 to the IL-6 receptor, with full inhibition being observed at roughly 4 µg/mL [117]. Tocilizumab has a long elimination half-life depending on the concentration. The mean elimination half-lives after 2, 4, and 8 mg/kg doses of tocilizumab were reported as 74.4, 96.9, and 160.2 h, respectively [115].

#### 2.3.4. Adverse Effects and Nutrition Interactions

The adverse effects of the greatest importance are skin and soft tissue infections, changes in liver function tests, hypercholesterolemia, neutropenia, and anaphylaxis [116]. Tocilizumab may cause moderate, reversible increases in triglyceride, high-density lipoprotein (HDL), low-density lipoprotein (LDL), and total cholesterol levels [118].

#### 2.3.5. Anakinra

Anakinra was the first biological drug to be developed as an interleukin IL-1 receptor antagonist (IL-1ra), having been derived from endogenous IL-1ra [119]. While it is very similar to IL-1ra, anakinra is not glycosylated. Furthermore, it possesses a residue of terminal methionine necessary for its biological production. The production of anakinra is undertaken using recombinant DNA technology from genetically modified Escherichia coli cultures [120]. This drug can block IL-1 activity in the synovial joints, which reduces the joint-destructive processes and inflammation seen in cases of rheumatoid arthritis [119]. Apart from rheumatoid arthritis, it has also been useful in treating various diseases ranging from gout and idiopathic pericarditis to hereditary diseases, such as familial Mediterranean fever [121].

#### 2.3.6. Mechanism of Action

Anakinra fully stops IL-1 from binding to the IL-1 type I receptor, blocking cell signaling and inhibiting the biological activities of IL-1 [122].

#### 2.3.7. Pharmacokinetics and Pharmacodynamics

Following subcutaneous dosing of 1–2 mg/kg of anakinra in patients with rheumatoid arthritis, maximum plasma concentrations occurred within 3–7 h and the elimination half-life was determined as 4–6 h [119]. The removal of anakinra from the body correlates directly with kidney function. In the case of mild or moderate renal impairment, decreases in the clearance of the drug do not have clinical relevance and dosage modification will not be required. For patients diagnosed with severe impairment or end-stage renal disease, however, dosage changes may be required [123].

#### 2.3.8. Adverse Effects and Nutrition Interactions

Anakinra is often linked to injection site reactions. A moderate increase in severe infections has been reported with the use of anakinra. Simultaneously, the combination of anakinra and etanercept (and possibly other TNF-α antagonists) increases infection risk and is, therefore, not recommended [124]. However, anakinra is a drug that increases insulin sensitivity. Evidence in this respect reveals the importance of IL-1 in the pathogenesis of insulin secretion and insulin resistance, which are defective in Type 2 diabetes [125].

### 2.4. Additional Treatments

#### 2.4.1. Azithromycin

Azithromycin is an antibiotic from the group of macrolides and it is used in the treatment of various bacterial infections [54]. The use of azithromycin together with hydroxychloroquine is recommended by the Ministry of Health of the Republic of Turkey for cases of possible/definitive COVID-19 or pneumonia with hospitalization indications and no complications [37,54].

#### 2.4.2. Mechanism of Action

Azithromycin is similar to the sugar portion of ganglioside GM1, which is a lipid acting as a host cofactor. Azithromycin can interact with the ganglioside binding site found on the SARS-CoV-2 protein, preventing the virus from binding to host cell receptors [126]. Azithromycin is known to be used as an adjunctive treatment in treating some viral respiratory infections for its anti-bacterial, anti-inflammatory, and immunomodulatory effects [72,127].

#### 2.4.3. Pharmacokinetics and Pharmacodynamics

Azithromycin undergoes rapid absorption following oral administration and distributes itself widely throughout the body, except in the cerebrospinal fluid. Peak plasma concentrations occur 2–3 h following administration of an oral dosage. Elimination half-life is 40–68 h. While protein binding is around 50% at very low plasma concentrations, it is lower at higher concentrations. Azithromycin is transformed into inactive metabolites by hepatic metabolism. The main elimination route is bile excretion, while urine excretes merely 12% of the drug unchanged. Absorption of only the capsule form, not the tablet or suspension form, decreases with food [128].

Azithromycin is preferred because it is a low-risk macrolide for CYP450-mediated drug interactions [11].

#### 2.4.4. Adverse Effects and Nutrition Interactions

Adverse effects include nausea, diarrhea, dyspepsia, flatulence, loss of appetite, dysgeusia, and abdominal cramps [54,73]. Taking azithromycin with nutrients reduces its absorption and results in a 43% reduction in its bioavailability [129]. It has been reported that it can interact with citrus fruits, citrus juices, and carbonated drinks. Bioavailability decreases due to acid variability as a result of the intake of nutrients [130].

#### 2.4.5. Corticosteroids (Methylprednisolone)

Corticosteroids and, especially methylprednisolone, are recommended as adjunct agents for treating COVID-19. Corticosteroids are commonly administered for the treatment of severe pneumonia and prevention of lung injuries thanks to their ability to suppress severe systemic inflammation. However, limited data have been reported regarding their use among COVID-19 patients [25].

In the pathophysiology of severe COVID-19, acute pneumonic processes, inflammatory infiltrates, extensive alveolar damage, and microvascular thrombosis are prominently observed [131]. Although a variety of therapeutic interventions are suggested by various sources to alleviate inflammatory organ damage in cases of viral pneumonia, the role of glucocorticoids is discussed with particular interest [132,133].

While small-scale studies have reported improvements in clinical outcomes with the usage of methylprednisolone in treating individuals diagnosed with COVID-19, the lack of reliable evidence from randomized, large-scale, clinical trials suggests the absence of any clear proof of the efficacy of glucocorticoids in these patients [134,135]. Corticosteroid therapy is not recommended routinely in cases of viral pneumonia due to fears that steroids may exacerbate lung injury [133].

Rapid deterioration of the clinical picture in cases of severe COVID-19 with viral pneumonia can progress to a disease similar to acute respiratory distress syndrome or even death as a result of ensuing multi-organ failure [136,137]. Heightened levels of interleukins and acute phase reactants as markers of systematic inflammatory response in COVID-19 patients have been reported, prompting clinicians to question the recommendations against corticosteroid use [138]. Even though there are guidelines stating that glucocorticoids are contraindicated in treatment or not recommended [139], the use of glucocorticoids is recommended by experts for severe cases in China [140]. A study was published in July 2020 revealing the positive effects of glucocorticoid usage for individuals diagnosed with COVID-19 in the United Kingdom and receiving mechanical ventilation support. However, that study reported no benefits from using corticosteroids for patients who did not need respiratory support [135]. In another study, a decrease was reported in the number of days without needing a ventilator after steroid administration [141].

#### 2.4.6. Mechanism of Action

The aim of corticosteroid use is to reduce the lungs’ inflammatory response, which may frequently escalate to acute lung injuries and acute respiratory failure syndrome. At the same time, though, they can also produce adverse effects, such as delayed viral clearance or heightened risks of secondary infections. Direct evidence supporting corticosteroid use in COVID-19 remains scanty at this time, but a review of the results obtained in other viral pneumonia cases may be helpful [133].

#### 2.4.7. Pharmacokinetics and Pharmacodynamics

The absorption of glucocorticoids is effective and they have 60–100% bioavailability [142]. Methylprednisolone displays protein binding rates of about 75%, primarily to transcortin and albumin. While insufficient pharmacokinetic data exist for methylprednisolone, it has been indicated that normal kidneys will eliminate 65–70% of this drug every day [143].

#### 2.4.8. Adverse Effects and Nutrition Interactions

Typical adverse effects related to the use of corticosteroids may be associated with energy metabolism and water, sodium, potassium, calcium, and phosphorus metabolism. Osteoporosis, cardiovascular diseases, impaired immune response, and glucose and lipid metabolism changes may occur [144]. High doses of corticosteroids are also linked in the literature with severe bacterial infections and hypokalemia [145]. When such complications occur, the diet therapy of the patients should be arranged according to these parameters.

#### 2.4.9. Convalescent Plasma Therapy

Convalescent plasma therapy is an important therapeutic approach for individuals diagnosed with COVID-19, and particularly for those in critical conditions [32]. Plasma taken from donors who have successfully recovered from COVID-19 may contain certain antibodies that can help suppress the virus and alter the inflammatory response. Many authoritative organizations such as the National Institutes of Health suggest that there are insufficient data values regarding the efficacy of convalescent plasma therapy for COVID-19 patients [146,147]. However, the FDA has announced its approval of Emergency Use Authorization for the production and administration of convalescent plasma for hospitalized patients with signs of infection after noting that the benefits of plasma therapy outweighed the risks among in-patients, considering the lack of other effective treatments [148]. The length of hospital stay, rate of transition to the ICU, and possible effects on mortality are among the issues that are still open to research [32]. Furthermore, there are no available data points on the impact of convalescent plasma therapy on nutritional status, hospital malnutrition, or specific nutrients. These issues must be explored in depth in future studies.

#### 2.4.10. Vitamins and Minerals

The chronic effects of the drugs being administered in the treatment of COVID-19 on long-term nutritional status and health are not known yet. In this regard, the nutritional deficiencies that may be caused by these agents in the long term are still among the issues that need to be clarified. However, there is some evidence suggesting that certain vitamins and minerals, which are reported to have supportive effects on the immune system, can increase the efficacy of these drugs in treatment [149,150,151].

#### 2.4.11. Vitamin D

Vitamin D directly exerts antiviral effects on enveloped viruses, and, since the coronavirus is also an enveloped virus, a link has been drawn between vitamin D levels and the prognosis of COVID-19. As a result of a systematic review, it was shown that serum vitamin D levels can serve as reliable indicators of the risk, severity, and mortality rate of COVID-19. Therefore, it is recommended to keep serum vitamin D at appropriate levels with supplements or adequate exposure to sunlight during the pandemic period [152].

In a randomized, placebo-controlled double-blind study of high doses of vitamin D3, participating subjects were divided into three groups: a placebo group, a group receiving 250,000 IU of vitamin D3, and a group receiving 500,000 IU of vitamin D3. It was determined that the group receiving 500,000 IU experienced significant elevations of hemoglobin concentration and had significantly decreased serum hepcidin concentrations over time as compared to the placebo group [153]. Furthermore, in a review recently conducted by Grant et al., it was advised that people with higher risk of influenza and/or COVID-19 infection take 10,000 IU/day for a few weeks, to be subsequently followed by 5000 IU/day, for a reduced risk of infection and to rapidly raise serum 25-hydroxyvitamin D [25(OH)D] concentrations to levels higher than 40–60 ng/mL or 100–150 nmol/L [154]. These recommendations were then criticized [155] and it was stated that the risks and benefits should be evaluated with more caution before administering mega-vitamin doses to COVID-19 patients since there is currently a notable lack of concrete evidence [149].

There are data values on the effects of various drugs used in the treatment of COVID-19 on serum vitamin D levels. It was shown in a meta-analysis study that the administration of corticosteroids can cause a decrease in vitamin 25(OH)D levels [156], while it was reported that Lop/r therapy for 48 weeks can result in significant elevations of serum 25(OH)D levels among patients [157]. Furthermore, it was reported that combinations of potential immunomodulatory compounds, such as vitamin D and remdesivir, may also offer better treatment results in the fight against COVID-19 and the possible synergistic effect between them should be examined [158].

#### 2.4.12. Vitamin C

COVID-19 pneumonia and the potential to progress to respiratory failure are thought to be caused by immune hyperreaction, which is a process in which IL-6 and endothelin-1 have key roles. Vitamin C has the capability of reducing inflammatory mediators. Accordingly, it may be beneficial to supplement vitamin C orally at a dosage of 1–2 g/day and with higher dosages in more severe cases. However, more clinical research is needed to obtain more conclusive evidence [150].

Considering that various drugs used for treating COVID-19 interact with vitamin C, taking corticosteroids together with vitamin C could improve oxygenation in patients with viral pneumonia treated in the ICU. However, it was also stated that those findings should be confirmed by larger studies [159]. It was stated that oseltamivir may also be used together with vitamin C in the treatment of individuals diagnosed with COVID-19 in the future due to its potential effects [160].

#### 2.4.13. Zinc (Zn)

Zinc may be effective in hampering the activity of the SARS-CoV-2 receptor ACE-2 in T-cell modulation and in reducing the cytokine storm that is observed in association with severe COVID-19 [151,161].

Zinc deficiency is known to cause humoral and cell-mediated immune dysfunction [162]. Among elderly populations, lower serum Zn levels of <0.7 mg/L are known to constitute a risk factor for pneumonia [163], while zinc deficiency increases inflammation and markers of inflammation over the long term [164]. In elderly patients, decreased serum zinc concentration has been found to correlate with increased cytokine levels (IL-6, IL-8, and TNF-α) [165].

Zinc supplementation used together with antiviral drugs such as hydroxychloroquine, ribavirin, remdesivir, or Lop/r in the treatment of individuals diagnosed with COVID-19 may support such a treatment [151]. Through in vitro studies, zinc has been demonstrated to successfully inhibit RNA-dependent RNA polymerase for SARS-CoV-2 [166]. It is challenging to create appropriate intracellular zinc concentrations with the sole application of prophylactic zinc against SARS-CoV-2, but in combination with a zinc ionophore like hydroxychloroquine/chloroquine. Cellular uptake increases together with the likelihood of achieving appropriately high intracellular concentrations [167,168].

Based on the therapeutic effects of hydroxychloroquine and chloroquine, their pharmacological effects as zinc ionophores, and the possible antiviral effects of zinc, it is thought that combining hydroxychloroquine/chloroquine with zinc in treating outpatients and/or inpatients with COVID-19 may help with the improvement of clinical outcomes and the reduction of mortality rates [17,169]. It was reported that zinc supplements do not alter the efficacy of hydroxychloroquine positively or negatively in clinical settings and they should be further examined with other drug therapies for COVID-19. As a preventive approach or treatment approach to COVID-19, zinc supplementation in combination with hydroxychloroquine/chloroquine is recommended for evaluation on a case-by-case basis [170]. Although the risk of side effects in zinc usage is low, its role in COVID-19 treatment still needs to be supported by clinical data. Zn-acetate, Zn-sulfate, Zn-gluconate, and Zn-picolinate can be used for zinc adjuvant therapy in forms with different amounts of Zn in each salt. It is important to remember that the optimal Zn dose varies in light of each individual’s age, sex, comorbidities, and general health status. Although Zn has positive effects on immune response, long-term supplementation of high doses of Zn may have effects such as increased LDL-cholesterol, anemia, and copper deficiency [171].

In summary, it is thought that zinc supplementation may have effects not only on the inflammation associated with COVID-19 but also on SARS-CoV-2 itself [172].

#### 2.4.14. Selenium (Se)

Selenium plays important roles in the protection of the respiratory system for various infectious diseases and especially against viral infections [173,174]. It is thought that selenium, along with vitamin E, is an essential cofactor in the enzyme group that tries to halt the formation of ROS and it may, thus, be protective against infections. Pathogens show higher rates of mutation in people with Se deficiency and that may contribute to the rapid evolution of pathogenic viral strains [175]. Therefore, researchers have suggested that Se deficiency might have an important role to play in the formation of SARS-CoV-2 [176]. A study conducted in China reported a relationship between the recovery rate of SARS-CoV-2 patients and their Se statuses [177]. Current studies support the hypothesis that Se might be related to SARS-CoV-2 infection and the course and clinical outcome of COVID-19 disease [178,179,180].

In light of these studies, it is clear that the effectiveness of some nutrients, such as vitamins and minerals, in COVID-19 treatment may change depending on the types and active ingredients of the drugs being used, as well as the therapeutic and adjuvant doses. Considering that most of the studies performed to date are supplementation studies, it is important to stress that these studies do not consider conditions similar to natural nutrition. Furthermore, it is clear that possible supplementation recommendations should be made by health professionals at appropriate doses, according to the individual’s health status in cases of absolute necessary.

In addition to the evidence regarding the effectiveness of vitamin-mineral supplementation in COVID-19 treatment, it is suggested that low serum levels of some vitamins and minerals may affect the overall treatment and, secondarily, the efficacy of the drugs with a negative impact on the course of treatment [150,172,179].

In summary, each drug potentially used in the treatment of individuals diagnosed with COVID-19 may cause some pharmacokinetic and pharmacodynamic changes in the human organism and potentially increase the risk of nutrition-drug interactions. In this regard, a summary is given in Table 2 to highlight some significant drug and nutrition interactions and to suggest some precautions for healthcare practitioners.

## 3. Conclusions

While the new coronavirus disease continues to spread rapidly around the world, there is still no fully proven pharmacological treatment method. Current treatment modalities are still in the trial phase. Studies have been ongoing or have aimed at reducing the symptoms of COVID-19, and these treatment modalities are often reported to cause some adverse effects and comorbidities. This is why the most striking result to emerge from this review is that, among those adverse effects, these drugs can potentially cause food-drug and nutrition-drug interactions. Such interactions, which have been frequently seen in COVID-19, can cause negative consequences in patient treatment, trigger adverse reactions, and negatively affect overall nutritional status rather than merely specific nutrients. The results of these interactions range from gastrointestinal symptoms to metabolic effects and alterations in the enzyme systems involved in food-nutrient-drug metabolism. Although a great number of these interactions have been reported in the currently available literature, it would not be surprising if, with further discovery of the disease and its potential effects, more cases of nutrition-drug interactions are eventually reported than are presently available. In addition, the pharmacokinetic and pharmacodynamic changes caused by these drugs used for supportive care can affect the metabolism of nutrients, both acutely and chronically, as should be expected. In addition, the administration of drugs and nutritional supplements, sometimes in the same way and especially in ICU patients and/or patients with insufficient oral intake, may increase the risk of this interaction pharmacodynamically.

Identifying food-drug interactions is as important as their management in terms of improving the health status of patients. Although this is not one of the priority targets at present, the importance of the chronic effects caused by food-drug interactions will increase over the course of the COVID-19 pandemic, which is currently expected to continue for a long time.

The severity, mechanisms, onset of action, and clinical significance of these interactions can vary individually. In this regard, especially with COVID-19, intestinal microbiota, nutrigenetic, and pharmacogenetic variations, and hormonal and metabolic factors that may cause food/nutrition-drug interactions or directly affect such interactions should be considered in future studies. More studies are needed to enlighten the cause-effect relationships between COVID-19 (as well as most common respiratory viruses) and nutrients as well as drugs, which are very different from simple associations, and where confounders cannot be accounted for.

Clinicians must pay close attention to the possibility of food-drug interactions when prescribing any new medications for treatment of individuals diagnosed with COVID-19. Additionally, as the existing evidence on food/nutrition-drug interactions increases during the COVID-19 pandemic, it is recommended to create protocols and diagrams for preventing food-drug interactions in the guidelines of authoritarian organizations on nutritional management for this disease.

## Figures and Tables

**Table 1:** Antiviral drugs, their mechanisms of actions, some adverse effects, and recommendations for administrations in treating COVID-19 infections [24,25,26,27,28].

Antiviral Drugs	Drug	Mechanism of Action	Most Common Adverse Effects	Administration via Feeding Tube
Viral entry blockers	Hydroxychloroquine	Increases the endosomal pH needed for the continuation of cell functions of the virus, further glycosylation of the cellular receptors of SARS-CoV-2 (ACE-2)	Common: Abdominal cramps, anorexia, nausea, diarrhea, vomiting Major: Cardiotoxicity, arrhythmia, hematopoietic system disease, hypoglycemia, neuropsychiatric and central nervous system effects, retinal toxicity	Splitting or crushing the film-coated tablet is generally not recommended. When necessary, the tablet can be crushed and mixed with water.
	Umifenovir	Spike protein/ACE-2 fusion inhibitor	Nausea and vomiting, less commonly, dizziness and psychiatric symptoms	
Viral RNA polymerase/RNA synthesis inhibitors	Remdesivir	Adenosine nucleotide analog, RdRp inhibitor, prodrug	Renal dysfunction Abnormal liver function tests	
	Favipiravir	Guanosine nucleotide analog, RdRp inhibitor, prodrug	Hyperuricemia, diarrhea, increased transaminases, decreased neutrophile numbers	The tablet can be crushed and mixed with water or juice.
	Ribavirin	Guanine analog, RdRp inhibitor	Hematopoietic system disease Dizziness Arterial pressure not decreased	
Viral protein synthesis inhibitors	Lopinavir/ritonavir	Protease inhibitor	Dizziness Cardiovascular system disease Arterial pressure not decreased Urinary system disease	Splitting or crushing the tablet is not recommended. When crushed, its bioavailability decreases. If the tablet needs to be crushed, the medicine should be placed in the syringe and 10 mL of water should be withdrawn. After 4 h of dissolving, it becomes a slurry and can be applied in this way.
Immunomodulators	Nitazoxanide	Interactions with regulated host pathways concerting viral replication, amplification of cytoplasmic RNA sensitivity, and type I IFN pathways	Abdominal pain, nausea, diarrhea, vomiting, headache	
Neuraminidase inhibitor	Oseltamivir	Prevention of viral spread in the human body, prodrug	Diarrhea, nausea, vomiting	The capsule can be opened. The content can be mixed into sweetened foods and drinks.
**Additional Treatments**				
Antibacterials applied in combination with hydroxychloroquine for synergistic antiviral action	Azithromycin	Prevention of viral binding among host cell receptors	Hematopoietic system disease Integumentary system disease Cardiovascular system disease	
Cytokine gene expression inhibitor	Corticosteroids (methylprednisolone)	Treatment of severe pneumonia and prevention of lung damage	Osteoporosis, cardiovascular diseases, impaired immune response, changes in glucose and lipid metabolism, stomach irritation, vomiting, headache, dizziness neuropsychiatric diseases, insomnia, dermatological problems	The tablet can be administered after dissolving in 10 mL of water.

SARS-CoV: Severe Acute Respiratory Syndrome Coronavirus-2. ACE-2: Angiotensin Converting Enzyme-2. RdRp: RNA-dependent RNA polymerase. IFN: Interferon.

**Table 2:** Some cautions and precautions of drug and nutrition interactions in COVID-19 for healthcare practitioners: a summary.

	Drugs	Nutritional Interactions and Potential Action Plan	References
**Antiviral Drugs**	Hydroxychloroquine/Chloroquine	Gastrointestinal symptoms such as nausea, diarrhea, anorexia, abdominal pain, vomiting Increased risk of hypoglycemia The nutritional status of the patient should be determined and recommendations should be made to encourage food intake and to control glycemia When cardiovascular problems like arrhythmia develop, cardioprotective nutrition programs should be arranged Patients with hereditary galactose intolerance, Lapp lactose deficiency, or glucose-galactose malabsorption problems are not eligible to use this drug. This patient group needs to be monitored for this reason.	[37,38]
	Favipiravir	Gastrointestinal symptoms such as diarrhea, nausea, and increased gas Increases in uric acid, ALT, AST, and blood triglyceride levels Decrease in neutrophils The nutritional status of the patient should be determined and recommendations should be made to encourage food intake A special nutrition plan should be adopted in the case of development of neutropenia.	[40,44]
	Remdesivir	Gastrointestinal symptoms such as nausea and vomiting The nutritional status of the patient should be determined and recommendations should be made to encourage food intake Increase in aminotransferase levels	[53,54]
	Lopinavir-Ritonavir	Diarrhea, nausea, vomiting, liver disorders, pancreatitis, neutropenia, hypercholesterolemia, and hypertriglyceridemia The nutritional status of the patient should be determined and recommendations should be made to encourage food intake. Appropriate dietary treatments should be planned by strict monitoring of other clinical values. St John’s wort (*Hypericum perforatum*) can cause a decrease in the plasma concentrations of the drug or a decrease in its clinical effects. The use of this herb in combination with this drug should be avoided.	[54,62,63,65]
	Umifenovir	Mild gastrointestinal adverse effects including nausea, diarrhea, and stomach pain Mild to moderate increases in ALT level The nutritional status of the patient should be determined and recommendations should be made to encourage food intake	[25,67]
	Oseltamivir	Concomitant intake with foods may delay the time to reach the highest density May cause impaired liver function tests, hepatitis, gastrointestinal bleeding, hemorrhagic colitis, diabetes exacerbation Taking it with food significantly reduces the adverse effects associated with the gastrointestinal system	[54,73,76]
	Ribavirin	It can lead to severe hemolytic anemia Rarely, symptoms of weakness and nausea may occur	[54]
	Nitazoxanide	Gastrointestinal symptoms such as diarrhea, oropharyngeal pain, abdominal pain, and vomiting may occur The nutritional status of the patient should be determined and recommendations should be made to encourage food intake When taken together with nutrients, its absorption is approximately doubled. Therefore, taking this drug with food rather than on an empty stomach can be encouraged, but the physician should be consulted on this issue for each patient.	[81,86,89]
**Anti-Inflammatory Drugs**	Ibuprofen	Gastrointestinal symptoms such as dyspepsia, nausea, and vomiting The nutritional status of the patient should be determined and recommendations should be made to encourage food intake	[101]
	Indomethacin	Adverse effects related to the cardiovascular, gastrointestinal, renal, and hepatic systems may occur. The persistence of these side effects over the long term will inevitably necessitate clinical nutrition therapy. Its absorption is reduced and delayed when taken with nutrients. However, its bioavailability is not affected.	[90]
**Interleukin Inhibitors**	Tocilizumab	Changes in liver function tests, neutropenia Moderate, reversible increases in total cholesterol, LDL, HDL, and triglyceride levels Appropriate diet therapy should be initiated to control dyslipidemia A special nutrition plan should be adopted in the case of development of neutropenia	[116,118]
	Anakinra	It may cause an increase in insulin sensitivity. Care should be taken for individuals who are treated to control glycemia.	[125]
**Some Additional Treatments**	Azithromycin	May cause gastrointestinal symptoms such as nausea, diarrhea, dyspepsia, flatulence, loss of appetite, abnormal taste changes, and abdominal cramps. In light of these findings, diet therapy should be planned and the food intake of the patients should be monitored. Taking it with nutrients reduces its absorption and bioavailability. Therefore, taking it on an empty stomach can be encouraged, but the physician should be consulted on this issue specifically for each patient. Its bioavailability decreases due to interaction with citrus fruits, citrus juices, and carbonated beverages. It should not be taken with such beverages (within 1–2 h).	[54,73,129,130]
	Corticosteroids (Methylprednisolone)	Changes in water, sodium, potassium, calcium, and phosphorus metabolism Osteoporosis, cardiovascular diseases, impaired immune response, and changes in glucose and lipid metabolism may occur Hypokalemia may occur with high doses of corticosteroids Appropriate diet therapy should be planned after thoroughly monitoring parameters regarding nutritional interactions	[135,136]

ALT: Alanine transaminase. AST: aspartate aminotransferase. LDL: low-density lipoprotein. HDL: high-density lipoprotein.

## Data Availability

Not applicable.
